# Relationship between Liver Mitochondrial Respiration and Proton Leak in Low and High RFI Steers from Two Lineages of RFI Angus Bulls

**DOI:** 10.1155/2015/194014

**Published:** 2015-04-23

**Authors:** G. Acetoze, K. L. Weber, J. J. Ramsey, H. A. Rossow

**Affiliations:** ^1^School of Veterinary Medicine, University of California, Tulare, CA 93271, USAuniversityofcalifornia.edu; ^2^Zoetis Inc., Kalamazoo, MI 49001, USA; ^3^School of Veterinary Medicine, University of California, Davis, CA 95616, USA

## Abstract

The objective of this research is to evaluate liver mitochondrial oxygen consumption and proton leak kinetics in progeny from two lineages of Angus bulls with high and low residual feed intake (RFI). Two Angus bulls were selected based on results from a genetic test for RFI and were used as sires. Eight offspring at 10-11 months of age from each sire were housed in individual pens for 70–105 days following a diet adaptation period of 14 days. Progeny of the low RFI sire had 0.57 kg/d (*P* = 0.05) lower average RFI than progeny of the high RFI sire. There was no difference in dry matter intake between low and high RFI steers, but low RFI steers gained more body weight (*P* = 0.02) and tended to have higher average daily gains (*P* = 0.07). State 3 and State 4 respiration, RCR, and proton leak did not differ between high and low RFI steers (*P* = 0.96, *P* = 0.81, *P* = 0.93, and *P* = 0.88, resp.). Therefore, the increase in bodyweight gain which distinguished the low RFI steers from the high RFI steers may be associated with other metabolic mechanisms that are not associated with liver mitochondrial respiration and proton leak kinetics.

## 1. Introduction

Residual feed intake (RFI) is defined as the difference between actual dry matter intake and dry matter intake regressed on average daily gain and midtest metabolic body weight [1]. Residual feed intake is a commonly used measure of feed efficiency in cattle and has been used to select more feed efficient bulls. However, a link between RFI and mitochondrial oxygen consumption or proton leak has not been shown and currently gene chips do not include any sequences for mitochondrial DNA that could be used for selection purposes. An understanding of the role of mitochondria in feed efficiency (RFI) and growth in cattle would aid in determining its importance in sire selection. Mitochondria produce most of the ATPs and so inefficiencies in mitochondrial energy conversion will profoundly impact energy production and efficiency. To date, only 3 research publications have explored the relationship between production, feed efficiency (RFI), and mitochondrial respiration in cattle. Brown et al. [2] compared liver mitochondrial respiration to estimated differences in heritability of milk production in Holsteins and growth and milk production in beef breeds. They found no correlation for beef breeds. Kolath et al. [3] were the first to measure skeletal muscle mitochondrial respiration in high and low RFI steers and found that low RFI steers had higher rates of mitochondrial respiration but mitochondrial function was not different. Proton leak kinetics could account for differences in mitochondrial respiration rates but was not measured in either study. However, Lancaster et al. [4] also found higher liver mitochondrial respiration rates in low RFI cattle in one of the two experiments but no difference in proton leak kinetics. Proton leak kinetics are used to represent the uncoupling of hydrogen ion passage with ATP production and to assess changes in mitochondrial respiration. For example, an increase in proton leak kinetics could increase mitochondrial oxygen consumption without increasing ATP production.

Liver is a highly active metabolic tissue and is the central organ of metabolism in the ruminant. Proton leak accounts for approximately 20% of total resting energy expenditure [5, 6] and is an important contributor to basal energy expenditure and net energy for maintenance. Therefore, it would be expected that liver mitochondrial respiration rates and lower proton leak kinetics would contribute to improved feed efficiency. The objective of this research is to evaluate liver mitochondrial respiration from progeny of two Angus bulls with high and low RFI to examine the association between RFI, liver mitochondrial respiration rates, and liver mitochondrial proton leak kinetics.

## 2. Material and Methods

### 2.1. Steers and Management

This experiment was approved by the University of California, Davis Animal Care and Use Committee. Two popular commercial Angus bulls were selected based on an observed difference in their genomic breeding values for RFI of 0.32 kg/d (Zoetis Inc., Kalamazoo, MI), placing the low RFI bull in the top 1% and the high RFI bull in the bottom 10% for the breed. These bulls were used to artificially inseminate a group of predominantly Angus cows at Sierra Foothill Research and Extension Center (Browns Valley, CA). From progeny produced in September 2011, eight steers per sire were selected for participation in the study. One low RFI steer was removed from the trial because it did not adapt to the feedlot environment. When steers were at the age of 10-11 months, they were shipped to UC Davis feedlot and, following 14 days of diet adaptation, were housed in individual pens. Pens were randomly reassigned every 14 days to avoid social effects on feeding behavior. Diet consisted of 62.6% rolled corn, 17.2% dry distillers grain, 7.83% alfalfa, 4.74% molasses, 3.91% oat hay, 1.96% fat, 1.28% limestone, 0.26% salt, 0.13% magnesium oxide, and 0.01% rumensin. The diet contained 1.76 MJ/kg NEm, 1.18 MJ/kg NEg, and 12.2% CP. Feed was individually weighed and provided 4 times daily. Body weights, hip heights, rectal temperatures for health assessment, and ultrasound backfat thicknesses were taken every 14 days. Slaughter criterion was established as a minimum of 11 mm of backfat thickness assessed by ultrasound [7]. Steers were fed for 70–105 d (September–January, 2013) and were slaughtered at the UC Davis slaughter facility where euthanasia was performed via captive bolt. Immediately after euthanasia, liver samples were collected and transported to the laboratory for mitochondrial isolation. Isolated mitochondria were immediately used for oxygen consumption and proton leak kinetics assays.

### 2.2. Mitochondrial Isolation

Approximately, 1 g of liver tissue was used for mitochondria isolation according to Cawthon [8, 9]. The tissues were minced in isolation media (220 mM mannitol, 70 mM sucrose, 20 mM Tris, 1 mM EDTA and 0.1% (w/v) BSA, pH 7.4 at 4°C). The minced tissue was homogenized in a Potter-Elvehjem vessel with a Teflon pestle of 0.16 mm clearance maintained on ice. The homogenate was centrifuged at 1,800 ×g for 10 min, and the resulting supernatant was centrifuged at 8,100 ×g for 10 min to obtain the mitochondrial pellet. Fatty acid free BSA was used in the isolation of mitochondria to scavenge free fatty acids that can induce or cause proton leak in the inner mitochondrial membrane and to act as a moderate free radical scavenger to prevent oxidation of lipids and proteins during the study. The pellet was resuspended and washed twice in 10 mL isolation solution with and without BSA at 8,100 ×g for 10 min each. The resulting mitochondrial pellet was suspended in 200 *μ*L of isolation medium and placed on ice for oxygen consumption and proton leak kinetics assays as described below. Protein concentration was determined using the Bradford protein assay with BSA as the standard.

### 2.3. Measurement of Mitochondrial Oxygen Consumption

Mitochondrial oxygen consumption was measured using a Hansatech Clark-type oxygen electrode (Norfolk, UK) [10, 11]. Mitochondria (1.0 mg protein/mL final concentration) were incubated in 1 mL of oxygen consumption medium (120 mM KCl, 5 mM KH_2_PO_4_, 5 mM MgCl_2_, 5 mM Hepes, and 1 mM EGTA) in a magnetically stirred incubation chamber maintained at 30°C. Rotenone (5 *μ*M) was used to block electron transport chain at Complex I and State 4 respiration (nonphosphorylating respiration) was determined in mitochondria following the addition of 5 mM succinate. State 3 respiration was measured in mitochondria incubated in the presence of 5 mM succinate and 100 *μ*M ADP. Respiratory control ratio (RCR) was determined by dividing State 3 by State 4 [12].

### 2.4. Measurement of Mitochondrial Proton Motive Force (Δ*p*)

Mitochondrial proton motive force (Δ*p*) [13] was assessed using a methyltriphenylphosphonium (TPMP^+^) sensitive electrode. All measurements were completed in duplicate and simultaneous to determinations of mitochondrial oxygen consumption. Rotenone (5 *μ*M) and oligomycin (8 *μ*g/mg protein) were used to respectively block electron transport chain at Complex I and ATP synthase. These chemicals raise membrane potential and ensure that all changes in oxygen consumption and membrane potential in response to sequential additions of malonate are due to proton leak.

Nigericin (0.4 *μ*g/mg protein) was added to convert the pH component of Δ*p* to membrane potential units (mV), allowing Δ*p* to be measured in mV units [11]. Data from the two electrodes (oxygen and TPMP^+^) were collected by data acquisition software (Hansatech Oxygraph System, Norfolk, UK) allowing real-time simultaneous measurements of mitochondrial oxygen consumption and Δ*p*. Mitochondrial membrane potential (MMP) in millivolts was calculated based on the Nernst equation [14] as follows:

 MMP = 61.5 log ([TPMP] added − external [TPMP]) × TPMP binding correction/(0.001 × mg of protein/mL × [TPMP]), where the TPMP binding correction was 0.4 (*μ*L/mg of mitochondrial protein)^−1^.

### 2.5. Statistical Analysis

All statistical analyses were performed using R Project for Statistical Computing (version 2.15.1). Data are presented as the mean ± SEM, and differences in means were detected using *t*-tests. Initial body weights, final body weights (BW), dry matter intake (DMI), average daily gain (ADG), liver weight, age at slaughter, days on feed (DOF), and average rectal temperature were tested as covariates and if significant were included in following model:

(1) $$\begin{array}{c} Y_{ij}=\mu +\alpha_{i}+\beta_{j}+\varepsilon_{ij}, \end{array}$$

where *Y*_*ij*_ = oxygen consumed (nmol/min) per mitochondrial protein (mg), *μ* is the overall mean, *α*_*i*_ is RFI group (*i* = 1,2), *β*_*j*_ is the covariate effect (*j* = 1,2,…, 7), and *ε*_*ij*_ are the residuals which follow a normal distribution *N*(0, *σ* ^2^).

A probability level of *P* ≤ 0.05 was considered statistically significant. For the analysis of proton leak kinetics, curves were estimated using the log function of Excel (Microsoft, 2007) and rates of oxygen consumption at a membrane potential of 150 mV were compared for high and low RFI steers using analysis of variance.

## 3. Results and Discussion

Mitochondrial DNA (mtDNA) is maternally inherited, explains 25–48% of variation in milk yield [2] and encodes 13 polypeptides involved in ATP synthesis [15]. However, the metabolic properties of mitochondria make them highly mutagenic environments. This mutational pressure introduces mtDNA variation (i.e., heteroplasmy) into the cytoplasmic population of cell lineages which can be influenced by sire genetics [16]. Thus selecting sires rather than dams for traits such as efficiency may be more beneficial to improve genetics associated with mitochondria metabolic activity. In this study, progeny of the low RFI sire had 0.57 kg/d (*P* = 0.05) lower average RFI than progeny of the high RFI sire. Therefore, progeny from both of the sires expressed a much greater difference in RFI than expected based on genomic predictions of 0.16 kg/d.

Only performance parameters relating to weight gain were different between low and high RFI steers (Table 1). Rectal temperatures were collected every 14 days and were not different between high and low RFI sires (*P* = 0.99). Age at slaughter, liver weight, and DMI also did not differ among progenies from high and low RFI sires (*P* = 0.69, *P* = 0.97, and *P* = 0.43, resp.). Initial body weights of steers entering the feedlot were not different, but final body weights at slaughter were greater in low RFI steers. Average daily gain also tended to be greater for the low RFI steers (*P* = 0.07). Therefore, the difference in RFI was due to increased gain and not changes in DMI. Unlike this study, Kolath et al. [3], Lancaster et al. [4], and Castro Bulle et al. [17] did not find differences in ADG or final body weight among high and low RFI steers but did observe that low RFI steers had smaller DMI compared to high RFI steers. Therefore, RFI from previous studies was based on differences in DMI. It would be expected that differences in DMI would be more likely to result in differences in mitochondrial oxygen consumption [3]. Differences in RFI due to gain may involve more post mitochondrial metabolic functions which would explain why mitochondrial oxygen consumption and proton leak kinetics were not different in high and low RFI steers in this study.

No differences in liver mitochondrial respiratory rates were observed between high and low RFI steers (Table 2). Oxygen consumption rates during State 3 respiration (maximum ADP stimulated respiration), State 4 respiration (leak-dependent respiration), and RCR did not differ between high and low RFI steers (*P* = 0.96, *P* = 0.81, and *P* = 0.93 resp.). Unfortunately only three other studies have been published relating production efficiency to mitochondrial respiration and function in cattle. Brown et al. [2] examined variability in mitochondrial respiration rates by measuring State 3, State 4, and RCR in livers from both Holstein lactating cows and beef cows (Angus, Brangus, and Hereford). Similar to this study, they did not find any correlation in mitochondrial respiration for beef cattle with growth or milking traits but did for Holstein milking traits. Kolath et al. [3] compared RFI in 16 Angus steers (9 low RFI steers and 8 high RFI steers) and State 2, State 3, and State 4 respiration rates and RCR in muscle mitochondria. But proton leak kinetics were not measured. Daily gain was not different between RFI groups, but DMI was greater and State 2 and State 3 respiration rates and RCR were lower for the high RFI steers. Similar to results from this study, mitochondrial function was not different between RFI groups, but in the Kolath et al. study [3] mitochondrial respiration rate was higher with low RFI steers. Therefore, mitochondrial respiration rate may be related to level of intake. The third study [4] examined liver mitochondrial respiration and proton leak kinetics and RFI in Angus heifers and Santa Gertrudis steers. Low RFI cattle had lower DMI but similar bodyweights and the same State 2 and State 4 respiration rates, proton leak kinetics, and RCR. State 3 respiration rates were higher for the low RFI Angus cows but were not different for the Santa Gertrudis steers. Therefore, it is unclear if differences among mitochondrial respiration rates, proton leak, and RCR exist for different species or tissues or are different with differences in feed efficiency [3, 4]. However, Bottje et al. [12] did report differences in RCR for leg and breast muscle in chickens, suggesting that mitochondrial respiration may differ among tissues.

Differences in feed efficiency were observed between low and high RFI steers in the present study. But, since no differences were found in liver mitochondrial respiration rates at State 3 and State 4 or in RCR values, the higher gain per kg of DMI of low RFI steers was not associated with a decrease in liver mitochondrial proton leak or the capacity for liver mitochondrial respiration. This may suggest that the size of the effect was too small to observe with this number of animals. However, Kolath et al. [3] did detect differences in respiratory rates with similar numbers of steers as in this study. Moreover, these results suggest that RFI of those two sire groups was not associated with liver mitochondrial respiration and proton leak kinetics and was instead driven by other cellular and physiological processes not measured in this study.

**Figure 1:**
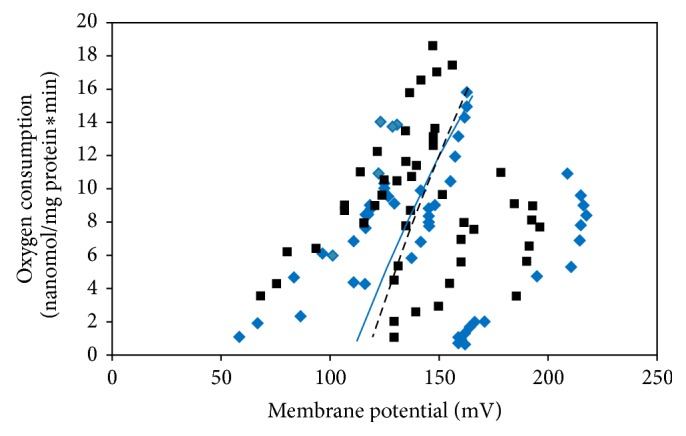
Liver mitochondrial proton leak kinetics for high (⧫) and low (■) residual fee intake Angus steers. Predicted values from log function for high (—) and low (**- - - -**) residual feed intake are represented by lines.

Mitochondrial proton leak is a process that dissipates proton motive force through the movement of protons across the mitochondrial inner membrane without production of ATP [18]. In the present study, there were no differences (*P* = 0.88) in hepatic mitochondrial proton leak, assessed by calculating rates of oxygen consumption at common membrane potential of 150 mV, in Angus steers with high and low RFI (Figure 1). Furthermore, State 4 respiration (leak-dependent respiration represented by the points on the* far right *of each curve) and membrane potential at State 4 respiration were the same for both RFI groups. Thus, liver mitochondrial proton leak was not different between the two groups of steers. However, correlation coefficients for regression analyses of log transformations using the Log Function of Excel were 0.82 and 0.63 for high and low RFI steers, respectively. These results agree with those of Lancaster et al. [4] and Bottje et al. [19] in which no differences in liver proton leak kinetics between high and low RFI steers and muscle basal mitochondrial proton leak were observed among high and low feed efficient broilers. The lack of differences in liver mitochondrial proton leak observed in this study indicates that feed efficiency is not associated with mitochondrial proton permeability, a major contributor to mitochondrial efficiency. Whether mitochondrial proton leak in other tissues is related to feed efficiency in beef cattle remains to be determined.

It was expected that differences in RFI between Angus steers were correlated with hepatic mitochondrial proton leak because proton leak is a major contributor to mitochondrial efficiency and resting energy expenditure [5, 6]. Thus, it was expected that less proton leak would be observed in low RFI steers due to increased energy partitioning towards gain. However, results of this study showed that energy partitioning between the two RFI groups was not the same. Energy intake was not different between low and high RFI steers, but differences were found for ADG. Therefore, we can conclude that low RFI steers were partitioning more energy towards gain, while high RFI steers were partitioning more energy towards other physiological processes.

## 4. Conclusions

Differences in low and high RFI beef steers were not associated with liver mitochondrial proton leak kinetics, a contributor to mitochondrial efficiency, or mitochondrial respiration rates (State 3 and State 4 respiration and RCR). Therefore, which tissues and biochemical processes are primarily responsible for differences in RFI in beef cattle remains to be determined.

## Figures and Tables

**Table 1:** Performance parameters of high and low residual feed intake (RFI) Angus bull progeny.

	Low RFI (*n* = 7)	High RFI (*n* = 8)	Mean difference	SEM^∗^	*P* value
Initial BW, kg	368.00	358.00	10.00	5.02	0.48
Final BW, kg	547.00	519.00	27.00	3.84	0.02
DMI, kg	8.53	8.51	0.01	0.16	0.97
ADG, kg	1.53	1.29	0.24	0.05	0.07
Liver weight, kg	6.01	5.99	0.01	0.13	0.97
RFI	−0.58	−0.01	−0.57	0.10	0.05
Age at slaughter, days	449.00	445.00	5.00	4.18	0.69

^∗^Pooled SEM (*n* = 7).

**Table 2:** Liver mitochondria respiration from high and low RFI Angus bull progeny.

	Low RFI (*n* = 7)	High RFI (*n* = 8)	SEM^†^	*P* value
State 3 respiration	31.30	30.80	9.42	0.95
State 4 respiration	9.76	10.40	3.23	0.80
RCR^∗^	3.05	3.03	0.24	0.93

^∗^RCR: respiratory control ratio (State 3 : State 4); State 3 and State 4 respiration data are presented as nanomoles of O_2_ consumed per milligram of mitochondrial protein per minute.

^†^Pooled SEM (*n* = 7).

## References

[1] Koch R. M., Swiger L. A., Chambers D., Gregory K. E. (1963). Efficiency of feed use in beef cattle. *Journal of Animal Science*.

[2] Brown D. R., DeNise S. K., McDaniel R. G. (1988). Mitochondrial respiratory metabolism and performance of cattle. *Journal of Animal Science*.

[3] Kolath W. H., Kerley M. S., Golden J. W., Keisler D. H. (2006). The relationship between mitochondrial function and residual feed intake in Angus steers. *Journal of Animal Science*.

[4] Lancaster P. A., Carstens G. E., Michal J. J., Brennan K. M., Johnson K. A., Davis M. E. (2014). Relationships between residual feed intake and hepatic mitochondrial function in growing beef cattle. *Journal of Animal Science*.

[5] Rolfe D. F. S., Brown G. C. (1997). Cellular energy utilization and molecular origin of standard metabolic rate in mammals. *Physiological Reviews*.

[6] Ku H.-H., Brunk U. T., Sohal R. S. (1993). Relationship between mitochondrial superoxide and hydrogen peroxide production and longevity of mammalian species. *Free Radical Biology and Medicine*.

[7] Williams A. R. (2002). Applications of ultrasound in livestock production systems: ultrasound applications in beef cattle carcass research and management. *Journal of Animal Science*.

[8] Cawthon D., McNew R., Beers K. W., Bottje W. G. (1999). Evidence of mitochondrial dysfunction in broilers with pulmonary hypertension syndrome (ascites): effect of t-butyl hydroperoxide on hepatic mitochondrial function, glutathione, and related thiols. *Poultry Science*.

[9] Cawthon D., Beers K., Bottje W. G. (2001). Electron transport chain defect and inefficient respiration may underlie pulmonary hypertension syndrome (ascites)-associated mitochondrial dysfunction in broilers. *Poultry Science*.

[10] Estabrook R. W. (1967). Mitochondrial respiratory control and the polarographic measurement of ADP:O ratios. *Methods in Enzymology*.

[11] Ramsey J. J., Hagopian K., Kenny T. M. (2004). Proton leak and hydrogen peroxide production in liver mitochondria from energy-restricted rats. *American Journal of Physiology—Endocrinology and Metabolism*.

[12] Bottje W., Tang Z. X., Iqbal M. (2002). Association of mitochondrial function with feed efficiency within a single genetic line of male broilers. *Journal of Poultry Science*.

[13] Ramsey J. J., Hagopian K., Kenny T. M. (2004). Proton leak and hydrogen peroxide production in liver mitochondria from energy-restricted rats. *The American Journal of Physiology—Endocrinology and Metabolism*.

[14] Rolfe D. F. S., Hulbert A. J., Brand M. D. (1994). Characteristics of mitochondrial proton leak and control of oxidative phosphorylation in the major oxygen-consuming tissues of the rat. *Biochimica et Biophysica Acta*.

[15] Sutarno J., Cummins J. M., Greeff J., Lymbery A. J. (2002). Mitochondrial DNA polymorphisms and fertility in beef cattle. *Theriogenology*.

[16] Rand D. M. (2001). The units of selection on mitochondrial DNA. *Annual Review of Ecology and Systematics*.

[17] Castro Bulle F. C. P., Paulino P. V., Sanches A. C., Sainz R. D. (2007). Growth, carcass quality, and protein and energy metabolism in beef cattle with different growth potentials and residual feed intakes. *Journal of Animal Science*.

[18] Ramsey J. J., Harper M.-E., Weindruch R. (2000). Restriction of energy intake, energy expenditure, and aging. *Free Radical Biology and Medicine*.

[19] Bottje W., Brand M. D., Ojano-Dirain C., Lassiter K., Toyomizu M., Wing T. (2009). Mitochondrial proton leak kinetics and relationship with feed efficiency within a single genetic line of male broilers. *Poultry Science*.

